# Development of a Virus-Like Particle-Based Anti-HER2 Breast Cancer Vaccine

**DOI:** 10.3390/cancers13122909

**Published:** 2021-06-10

**Authors:** He Hu, Nicole F. Steinmetz

**Affiliations:** 1 Department of NanoEngineering, University of California San Diego, 9500 Gilman Dr., La Jolla, CA 92039, USA; Aandyh20@vt.edu; 2 Department of Bioengineering, University of California San Diego, 9500 Gilman Dr., La Jolla, CA 92039, USA; 3 Department of Radiology, University of California San Diego, 9500 Gilman Dr., La Jolla, CA 92039, USA; 4 Center for Nano Immuno-Engineering, University of California San Diego, 9500 Gilman Dr., La Jolla, CA 92039, USA; 5 Moores Cancer Center, University of California San Diego, 9500 Gilman Dr., La Jolla, CA 92039, USA; 6 Institute for Materials Discovery and Design, University of California San Diego, 9500 Gilman Dr., La Jolla, CA 92039, USA

**Keywords:** virus-like particle, cancer vaccine, HER2, CpG-ODN, breast cancer

## Abstract

**Simple Summary:**

Virus-like particles (VLPs) have attracted significant interest as immunotherapy platforms and cancer vaccines for inducing antigen-specific immune responses against tumors. We prepared a human epidermal growth factor receptor-2 (HER2) cancer vaccine, by conjugating the HER2-derived CH401 epitope to the external surface of Physalis mottle virus (PhMV)-like particles via copper-free click chemistry. Another candidate was prepared by loading Toll-like receptor 9 (TLR9) agonists as adjuvant into the interior cavity of PhMV-CH401—although the addition of the adjuvant conferred no additional immune priming. The VLP-based anti-HER2 vaccine candidate was administered subcutaneously, using a prime-boost immunization schedule and BALB/c mice. The vaccine candidate elicited a strong immune response, including high titers of HER2-specific immunoglobulins and increased the toxicity of antisera to DDHER2 tumor cells. DDHER2 tumor challenge studies demonstrated efficacy, as evident from the delayed onset of tumor growth and the prolonged survival of the vaccinated vs. naïve BALB/C mice.

**Abstract:**

To develop a human epidermal growth factor receptor-2 (HER2)-specific cancer vaccine, using a plant virus-like particle (VLP) platform. Copper-free click chemistry and infusion encapsulation protocols were developed to prepare VLPs displaying the HER2-derived CH401 peptide epitope, with and without Toll-like receptor 9 (TLR9) agonists loaded into the interior cavity of the VLPs; Physalis mottle virus (PhMV)-based VLPs were used. After prime-boost immunization of BALB/c mice through subcutaneous administration of the vaccine candidates, sera were collected and analyzed by enzyme-linked immunosorbent assay (ELISA) for the CH401-specific antibodies; Th1 vs. Th2 bias was determined by antibody subtyping and splenocyte assay. Efficacy was assessed by tumor challenge using DDHER2 tumor cells. We successful developed two VLP-based anti-HER2 vaccine candidates—PhMV-CH401 vs. CpG-PhMV-CH401; however, the addition of the CpG adjuvant did not confer additional immune priming. Both VLP-based vaccine candidates elicited a strong immune response, including high titers of HER2-specific immunoglobulins and increased toxicity of antisera to DDHER2 tumor cells. DDHER2 tumor growth was delayed, leading to prolonged survival of the vaccinated vs. naïve BALB/C mice. The PhMV-based anti-HER2 vaccine PhMV-CH401, demonstrated efficacy as an anti-HER2 cancer vaccine. Our studies highlight that VLPs derived from PhMV are a promising platform to develop cancer vaccines.

## 1. Introduction

Virus-like particles (VLPs) have shown exceptional promise as vaccines and immunotherapies targeting cancers and infectious diseases, amongst other target areas [1]. VLPs are protein structures isolated from plant or mammalian viruses or bacteriophages, assembled in the absence of genomic nucleic acid (albeit several VLPs will package host nucleic acids to some degree) [2]. While non-infectious, the repetitive proteinaceous structure of the VLPs mimic pathogen-associated molecular patterns (PAMPs), making them highly visible to the immune system [1]. Therefore, the VLPs themselves function as an adjuvant and stimulate the immune system to improve vaccine efficacy. Based on their size, VLPs promote antigen localization to dendritic cell-enriched draining lymph nodes and enhance endocytosis of antigens through the antigen presenting cells (APCs); they then stimulate APCs via PAMPs recognition, and therefore, increase antigen presentation to the adaptive immune cells [2]. Moreover, with the well-defined structural knowledge, the VLPs are not only an adjuvant but also a delivery agent, offering high-precision nano-chemistry and an ideal scaffold for vaccine design through chemical or genetic engineering methods [1]. Based on these principles, various antigens have been conjugated or displayed on VLPs for their use as vaccines; and several systems have been or are currently in investigation in clinical trials [3,4,5,6,7,8,9].

Breast cancer remains the most common cancer (30%) among women’s cancers and has a significant mortality rate according to the 2021 statistics from the American Cancer Society [10]. Human epidermal growth factor receptor-2 (HER2) is overexpressed in 20–30% of breast cancer and is associated with aggressive tumors, a high rate of metastasis, and poor prognosis [11,12]. Therefore, HER2 is one of the most promising tumor-associated antigens (TAAs) of breast cancer and can be an attractive target for the development of immunotherapy [11]. Accordingly, the monoclonal antibody (mAbs), such as Trastuzumab (Herceptin) [13] and Pertuzumab (Perjeta) [14], and kinase inhibitors such as Laptinib, combined with neoadjuvant chemotherapy, have dramatically increased survival rates in patients with HER2+ breast cancer [15]. However, serum sickness, cardiac dysfunctions, and development of drug resistance to HER2-specific therapeutic antibodies remain as challenges to be overcome [16]. Moreover, passive immunotherapy does not protect against relapse, and given the short half-life of mAbs, large doses and frequent administrations are required [17]. Active immunotherapy through cancer vaccines holds the potential to overcome these shortcomings [18]. The challenge to develop effective anti-HER2 vaccines is to overcome tolerance to the self-antigen; this can be achieved through targeted delivery of antigenic epitopes, along with a potent adjuvant to APCs [19]. Indeed, research has shown that conjugation of HER2-derived epitope peptides to an antigenic carrier can induce antibodies that recognize HER2 expressed on tumor cells [1,2,20]; and several anti-HER2 peptide vaccines, such as E75 [21], GP2 [22], and AE37 [23] formulations have advanced into clinical testing.

Miyako et al. identified the CH401 epitope from the human HER2 extracellular domain (N: 167–175). CH401 contains epitopes for B cell and helper T cells, and an anchoring motif of the MHC class II molecule [24]. Our group previously reported the development of VLPs as carriers for CH401 and application as anti-HER2 cancer vaccines [25,26,27]. For example, we have compared the structure–function relationship of vaccine efficiency between two distinct VLPs platforms, by using the icosahedral cowpea mosaic virus (CPMV) and filamentous potato virus X (PVX), as carriers to deliver the CH401 peptide [25]. Our results have shown that the icosahedral CPMV significantly enhanced lymphatic drainage and immune cell interactions, as compared to filamentous PVX. Based on these results, we further studied the vaccine efficacy of the CPMV-CH401 in different HER2+ tumor models, including ectopic and orthotopic primary tumor and metastatic tumor in mice [26]. The results illustrated that the VLP-based vaccine candidate efficiently primed the development of HER2-sepcific antibodies, as well as effector and memory T cells, which contributed to delayed onset and progression of both primary and metastatic tumors, resulting in prolonged survival. Lately, we also developed a heterologous prime-boost strategy to deliver the CH401 epitope, using a set of distinct VLPs—CPMV, cowpea chlorotic mottle virus (CCMV) and Sesbania mosaic virus (SeMV) [27]. The three vaccines were administered sequentially as prime-boost, and each candidate was used only once; this strategy conferred higher titers of HER2-specific immunoglobulins vs. carrier-specific antibodies (which are produced after frequent administration of the same carrier), increasing the toxicity of the antisera toward cancer cells, and prolonging the survival of mice challenged with DDHER2 tumor cells. Overall, our previous studies have demonstrated efficacy and safety of the VLP-based vaccine candidates. This approach induced potent and long-lasting immune responses against the target antigen by efficiently targeting APCs, priming innate and adaptive immune responses, leading to potent antitumor efficacy. The heterologous prime-boost study also highlights the need for the development of a library of VLP-based vaccine candidates that could be combined as sequential doses.

Building on this work, here we set out to study a different VLP; namely the VLPs based on Physalis mottle virus (PhMV). The current COVID-19 pandemic highlights the need for vaccine platform technologies. There is no one-fits-all solution, therefore, there is a need to develop vaccine platform technologies. Other plant VNPs have been utilized—however, each platform is distinct, therefore, PhMV provides a novel technology that has not yet been utilized for chemical peptide epitope display. In previous works, we have systematically investigated this platform and demonstrated biocompatibility [28], and documented the in vivo biodistribution [29] and pharmacokinetic profile [29]. Our previous work focused on the application of PhMV for imaging and drug delivery [30,31]. Here, we explored the development of PhMV as nanovaccine candidate for treatment of HER2+ breast cancer. Two formulations were designed. (1) A VLP displaying the CH401 antigen, specifically, the rat-derived CH401 epitope 163YQDMVLWKDVFRKNNQLAPV182 was developed and termed PhMV-CH401. Copper-free click chemistry was used to conjugate the peptide epitope to surface-exposed Lys side chains on the PhMV VLP. (2) A VLP displaying the CH401 antigen on its surface and loaded with a Toll-like receptor 9 (TLR-9), agonist, specifically synthetic oligodeoxynucleotides (ODNs) containing unmethylated CpG motifs was developed and termed CpG-PhMV-CH401. CpG-ODN was added as an additional adjuvant; CpG-ODN trigger innate immune cell activation through TLR9 signaling, and in particular, activate human plasmacytoid dendritic cells (pDCs) and B cells [32]. Preclinical studies provide evidence that CpG-ODNs are potent adjuvants for vaccines targeting infectious disease and cancers [33]. Ongoing clinical studies indicate that CpG-ODNs are safe in humans and increase the immunogenicity of co-administrated vaccines [32,33]. Codelivery of the CpG-ODNs with nanoplatforms such as lipid, polymer, G-rich DNA ligands, gold nanoparticles, and VLPs, could enhance chemical stability and delivery efficacy [34]. These strategies overcome the short half-life and unfavorable pharmacokinetic profiles of the CpG-ODNs in vivo [34]. For these reasons, we developed the two vaccine formulations, one with and the other without the additional adjuvant. Specifically, we used the A-type mouse-specific TRL9 agonist CpG-ODN 1585, because it has been shown to stimulate and mature pDCs, leading to IFN-α secretion, which is expected to boost immunity against the target antigen codelivery [33]. We developed an encapsulation strategy and click-chemistry protocols; the vaccine candidates were then tested using the DDHER2 murine model of HER2+ breast cancer. Vaccine efficacy and underlying immunological mechanism were investigated.

## 2. Materials and Methods

### 2.1. Preparation of VLPs and Vaccine Formulation 

VLPs were prepared by expressing PhMV coat protein (CP) subunits in *E. coli*, and purified as reported [29,35]. For the loading of CpG-ODN into PhMV VLPs, purified VLPs were lyophilized and redispersed in D.I. water, followed by treatment with RNase A to remove the residual host RNA; we previously reported this as an effective method to remove RNA from CPMV [36]. The rat-derived HER2 epitope CH401 (sequence 163YQDMVLWKDVFRKNNQLAPV182-GPSL-N_3_, representing amino acid residues 163–182) were conjugated to the external surface of the VLPs, using copper-free click chemistry. In brief, 1 molar equivalents per coat protein (Eq/CP) of DBCO-PEG_4_-NHS reacted with 2 mg/mL VLPs in 0.1 mM pH 7.0 HEPES buffer at room temperature, for 3 h; the excess reagent was removed by ultracentrifugation (116,525 g, 1 h, room temperature) over a 30% (*w*/*v*) sucrose-cushion. The obtained PhMV-DBCO were redispersed in HEPES buffer and mixed with 1 Eq/CP of CH401-N_3_, gently shaking overnight at room temperature. The PhMV-CH401 particles were then purified again using the ultracentrifugation method. For loading the CH410-labeled VLPs with CpG-ODN 1585 (sequence Cy5.5-5′- ggGGTCAACGTTGAgggggg), 100 μM Cy5.5-CpG ODN 1585 per mg of PhMV-CH401 was incubated in 1 × PBS (pH 7.4) at 37 °C overnight. The products were purified through ultracentrifugation and washed twice using PBS. All final products were dispersed and stored in 1 × PBS (pH 7.4). VLPs were characterized by UV/Vis spectroscopy, native and denaturing gel electrophoresis, zeta potential, size exclusion chromatography (SEC), and transmission electron microscopy (TEM), as described in the Supporting Information.

### 2.2. Immunization

Animal experiments were carried out according to IACUC-approved procedures at the University of California San Diego. BALB/c (female, 6 weeks of age) were immunized subcutaneously on days 0, 14, and 28, with the CpG-PhMV-CH401 and PhMV-CH401 vaccines (100 μg per immunization, *n* = 8); free CH401 peptide (2.3 μg) and PhMV particles (100 μg) were used as the control groups (*n* = 3). Blood was collected in Greiner Bio-One VACUETTE^TM^ MiniCollect^TM^ tubes (Thermo Fisher Scientific, Waltham, MA, USA), through retro-orbital bleeding, prior to the first injection, i.e., day 0, and on day 35. The serum was separated through centrifugation at 10,000× *g* for 10 min, then stored at 4 °C, until analysis.

### 2.3. Enzyme-Linked Immunosorbent Assay (ELISA)

The pre-sera and the sera collected on day 35 were analyzed by ELISA to determine the antibody titers and isotypes. To quantify antibody binding to CH401, a cysteine-terminated CH401 peptide (CH401-GPSL-Cys) was dissolved in a coating buffer (10 × PBS with 10 mM EDTA, pH 7.2), at a concentration of 4 μg/mL, and 100 μL was added per well to 96-well Pierce^TM^ Maleimide Activated Plates (Thermo Fisher Scientific), followed by overnight incubation at 4 °C. The plates were then blocked with 10 μg/mL cysteine (100 μL/well), for 1 h at room temperature. After washing the plates with 0.05% (*v*/*v*) Tween-20 in PBS (washing buffer), the sera were diluted with coating buffer in two-fold serial dilutions, starting from 1:100, and were added to the plates in triplicates (100 μL/well). After incubating for 2 h at room temperature, the plates were washed with washing buffer four times and then incubated with alkaline phosphatase-labeled goat anti-mouse IgG (Invitrogen, Thermo Fisher Scientific) diluted 1:2000 in PBS (100 μL/well), for 1 h at room temperature. After washing, 1-step PNPP substrate (Thermo Fisher Scientific) was added (100 μL/well) and the reaction was stopped after 15 min, by adding 2 M NaOH (50 μL/well). The absorbance at 405 nm was then determined using a Tecan microplate reader.

VLP carrier-specific IgG and isotypes levels were similarly determined using 96-well PolySorp plates (Thermo Fisher Scientific) coated with 2 μg/well of PhMV. The plates were blocked with PBS containing 1% (*w/v*) BSA. The mouse sera and the alkaline phosphatase-labeled goat anti-mouse IgG were also diluted in PBS with 1% (*w/v*) BSA. The other steps were the same as described above for the epitope ELISA.

The distribution of IgG1, IgG2a, IgG2b, IgG3, and IgM in sera was determined by ELISA, using a similar procedure to that described above. Detection was carried out using horseradish peroxidase (HRP)-labeled goat anti-mouse IgG1 and IgG2a (Thermo Scientific), IgG2b, IgG3, and IgM (Abcam) secondary antibodies (1:2000), incubated for 1 h at room temperature. After washing the plates, 1-step^TM^ ultra TMB substrate (Thermo Fisher Scientific) was added (100 μL/well) and the reaction was stopped after 15 min, by adding 2 M H_2_SO_4_ (50 μL/well). The absorbance at 450 nm was then determined using a Tecan microplate reader.

### 2.4. Flow Cytometry

The mouse HER2+ cell line DDHER2 was a gift from Dr. Darrel Irvine’s laboratory at MIT, Cambridge-MA. DDHER2 cells were maintained in DMEM (Corning Life Sciences, Durham, NC, USA) supplemented with 10% (*v*/*v*) fetal bovine serum and 1% (*w*/*v*) penicillin/streptomycin. The cells were incubated at 37 °C in a 5% CO_2_ atmosphere. DDHER2 cells were harvested during the logarithmic growth phase in enzyme-free Hank’s-based Cell Dissociation Buffer (Thermo Fisher Scientific). The cells were then washed with FACS buffer (PBS containing 1% (*v*/*v*) fetal bovine serum) and resuspended in 200-μL FACS buffer, in a V-bottom 96 plate at 500,000 cells/well, in triplicates. The cells were then incubated for 1 h at 4 °C with mouse serum diluted 1:300 in FACS buffer. After washing the cells twice with FACS buffer, the cells were stained with an Alexa Fluor 647-labeled goat anti-mouse IgG antibody (Thermo Fisher Scientific), with 1:1000 dilution in FACS buffer. The cells were again washed twice with FACS buffer and resuspended in 500-μL FACS buffer for immediate analysis, using a BD Accuri™ C6 plus flow cytometer.

### 2.5. Complement-Dependent Cytotoxicity

DDHER2 cells were cultured and harvested as described above. The cells were washed with PBS and resuspended in 200 μL FACS buffer in a 1.5 mL Eppendorf tube, at 80,000 cells per tube. The cells were then incubated for 1h at 4 °C with mouse serum diluted 1:50 in FACS buffer. After washing three times with PBS, the cells were resuspended in a 200 μL serum-free medium and transferred to 96 well plates in four replicates (50 μL/well, 20,000 cells per well for DDHER2). Rabbit C12CC complement (BioRad) was diluted 1:20 in a serum-free medium and added to the plate (50 μL/well). Rabbit complement inactivated by heating at 65 °C for 30 min was incubated with cells that were not exposed to mouse serum; this sample served as a control representing 100% cell viability. Cells were incubated at 37 °C with 5% CO_2_ for 4 h, before adding 100 μL per well of 0.5% (*w*/*v*) methylthiazo-yldiphenyl-tetrazolium bromide (MTT) in PBS. After incubation at 37 °C for 2 h, the solution was carefully removed and 100 μL per well of DMSO was added, before measuring the absorbance at 490 nm on a Tecan microplate reader.

### 2.6. Cytokine Assay

IFN-γ and IL-4 secreted by primary splenocytes were detected using cytokine kits from Thermo Fisher Scientific, based on the manufacture’s protocol. The splenocytes were obtained from three immunized mice per group on day 35 (7 days after the third immunization). The mice were euthanized and the isolated spleens were sliced into small pieces and pressed through a 70 μm cell strainer (BD). The cells were washed with PBS and then the red cells were lysed. The splenocytes were resuspended in a T-cell medium (without supplement of IL-4) at 4 × 10^6^ cells/mL, and were transferred to 24-well plates (1.5 mL/well). The cells were incubated for 18 h at 37 °C in a 5% CO_2_ atmosphere, with and without the CH401 peptide (20 μg/mL), and the supernatants were collected for the cytokine ELISA kits to quantify the levels of IFN-γ and IL-4.

### 2.7. In Vivo Tumor Challenge

DDHER2 cells were cultured and harvested as described above. After washing with PBS, the cells were resuspended in a medium at 2 × 10^7^ cells/mL and mixed 1:1 with Matrigel (Corning Life Sciences). A total of 100 μL of the mixture (1 × 10^6^ cells) were subcutaneously injected into the right flank of each mouse, 14 days after the third immunization (*n* = 5). Tumor volumes were monitored and recorded every other day. Tumor size and body weight were measured and tumor volume was calculated using the formula ν =$\;l\times w^{2}/2$. Mice were euthanized if the tumor size reached 1000 mm^3^ according to the IACUC guidelines.

## 3. Results and Discussion

### 3.1. Synthesis and Characterization of the VLP-Based HER2 Vaccine Candidates

PhMV-based VLPs were prepared by expressing the PhMV coat protein in *E. coli*, as reported previously [28,35]. The assembled VLP formed a ~30 nm-sized icosahedron comprising 180 identical coat protein units. The crystal structure indicated that four Lys side chains (K62, K143, K153, and K166) were exposed on the external surface of each coat protein, offering 720 potential conjugation sites. Purified PhMV VLPs were freeze dried by negative pressure lyophilization to dehydrate the VLPs and eject the RNA, based on our previous method [36]. The obtained powder was redispersed in D.I. water and then the VLPs were treated with RNase A to remove any packaged RNA. We hypothesized that the loading of CpG-ODN would be most efficient after removal of any nucleic acids; and indeed, the loading of Cy5.5-labeled CpG-ODN 1585 was more efficient when using VLPs prepared by lyophilization, followed by RNase treatment step (see Figure S1).

**Figure 1:**
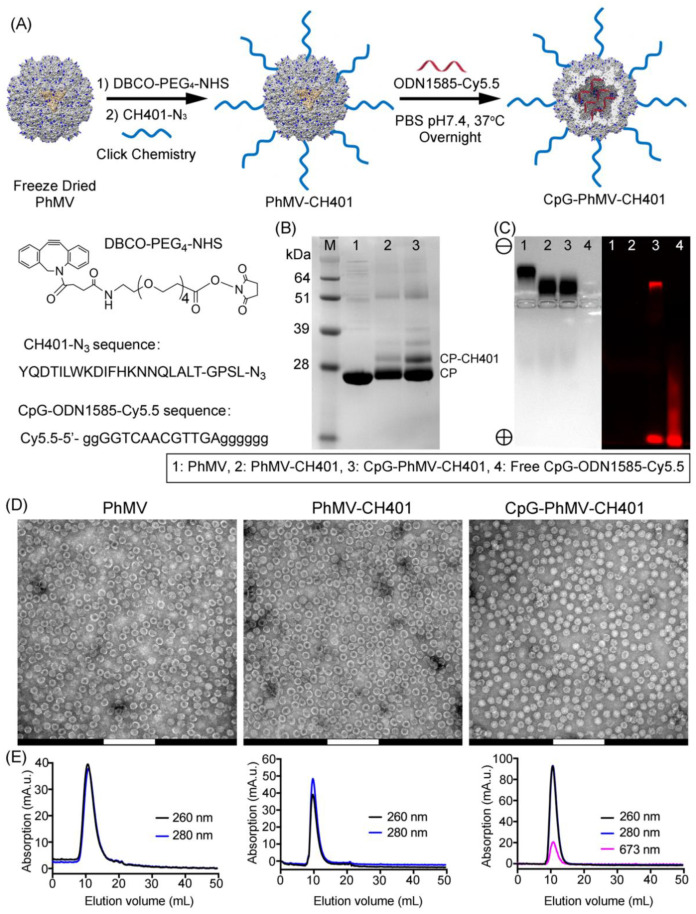
Synthesis and characterization of the PhMV-CH401 and CpG-PhMV-CH401 vaccine candidates. (**A**) Conjugation scheme of the HER2 epitope CH401 (rat) designed with a flexible GPSL linker and C-terminal azide was conjugated to the amine residues (from Lys side chains) of PhMV via a hetero-bifunctional *N*-hydroxysuccinimide PEG_4_-DBCO linker. To this end, copper-free click chemistry was used and it was loaded with fluorescence dye Cy5.5-labelled CpG-ODN 1585, to produce PhMV-CH401 and CpG-PhMV-CH401 vaccine candidates, respectively. (**B**) SDS-PAGE analysis of the VLPs followed by staining with Coomassie Brilliant Blue; CP—capsid protein. (**C**) Analysis of the VLPs through agarose gel electrophoresis, followed by staining with Coomassie Blue (left) and fluorescence imaging (right). (**D**) Transmission electron micrographs (TEM) of negatively-stained VLPs (Scale bar = 200 nm) and (**E**) size-exclusion chromatograms (SEC) of native PhMV and VLP-based vaccines.

Two vaccine candidates were formulated with CH401 displayed on the exterior surface of the VLPs, with or without CpG-ODN 1585 loaded into the VLP’s cavity (Figure 1A). The CH401 epitope 163YQDMVLWKDVFRKNNQLAPV182 (derived from rat HER2 protein, amino acid residues 163–182) was synthesized with a C-terminal flexible GPSL linker and a C-terminal azide group, enabling a click reaction to alkyne-functionalized PhMV. The alkyne-handle was introduced using a bifunctional *N*-hydroxysuccinimide PEG_4_-DBCO linker. Cy5.5-labeled CpG-ODN 1585 were added post CH401 conjugation. Purified PhMV-CH401 and CpG-PhMV-CH401 were characterized by SDS-PAGE (Figure 1B) and agarose gel electrophoresis (Figure 1C). SDS-PAGE analysis was consistent with covalent modification of the VLPs, as evident by a second, higher molecular weight band at ~29 kDa, in addition to the native PhMV capsid protein band at ~26 kDa; the higher molecular weight band corresponded to CP-CH401 (CH401-PEG_4_-, Mw~3.6 kDa). Densitometric analysis of the protein bands, using an AlphaImager^®^ gel documentation system, indicated conjugation of ~20% of the VLPs’ coat proteins; or in other words, conjugation of ~36 CH401 peptides per particle (equivalent to 2.3 ng/mg of protein). Native agarose gels were analyzed by fluorescence imaging and under white light, after staining with Coomassie Brilliant Blue. Imaging data showed that the native VLPs migrated toward the cathode, consistent with their positive charge (+4.5) [29]. However, this was affected by the presence of CH401 peptides, which are negatively charged, and the loading of CpG-ODN, which is also strongly negatively charged, causing migration of PhMV-CH401 and CpG-PhMV-CH401 toward the anode (Figure 1C). Two fluorescent bands were observed for the CpG-PhMV-CH401 formulation; one matching the protein-stained band indicating Cy5.5-labled CpG-ODN 1585 comigrated with the VLPs—thus, was stably loaded into or adsorbed onto the VLPs—and one high mobility band (toward the anode), which represented free CpG-ODN 1585. As free CpG-ODN 1585 was not observed by SEC (see below), we hypothesized that the VLPs were stably loaded into or absorbed onto CpG-ODN 1585 under native conditions. However, some fraction of the CpG-ODN 1585 was bound more weakly and could dissociate during electrophoresis. To quantify the CpG-ODN 1585 per VLP, we made use of the Cy5.5 label. The protein concentration of CpG-PhMV-CH401 was determined using the BCA protein quantitation assay kit and the concentration of CpG-ODN 1585 was determined by UV/visible spectroscopy and calculated using the Beer−Lambert law and Cy5.5 extinction coefficients. The assays indicated that ~23 CpG-ODN 1585 agonists were loaded per VLP, which was equivalent to ~4.9 nmol CpG-ODN 1585 per mg of protein.

The stability of VLP formulations after modification was characterized by TEM and SEC. The TEM images (Figure 1D) showed both unmodified PhMV VLPs and modified PhMV-CH401 and CpG-PhMV-CH401 vaccine candidates as regular nanoparticles measuring ~30 nm in size, aggregates or broken particles were not detectable. SEC revealed a single peak eluting at ~7.8 mL for the protein component (A_260/280_) and a corresponding peak for Cy5.5 (A_673_), indicating that the CpG-ODN 1585 was encapsulated in the CpG-PhMV-CH401 particles (Figure 1E); there was no evidence of free CpG-ODN 1585 by this method. Together, this data confirmed that both vaccine candidates yielded stable formulations with the VLPs, keeping their structural integrity.

### 3.2. Immunogological Assessment of the VLP-Based HER2 Vaccine Candidates

**Figure 2:**
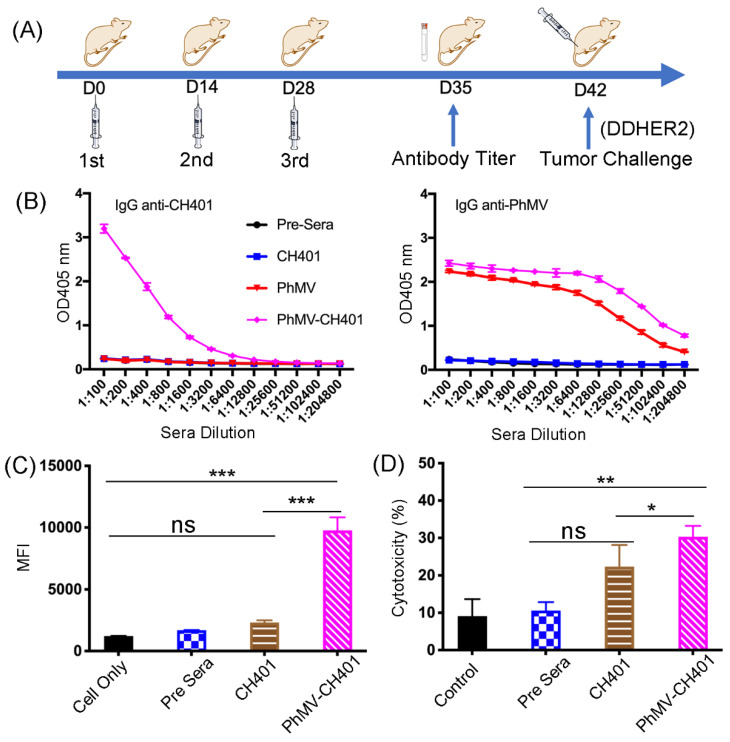
Immunological evaluation of the PhMV-based vaccines. (**A**) Immunization schedule using female BALB/c mice and subcutaneous administration of the vaccine candidates and controls; sera were collected before and after immunization, and the mice were challenged with DDHER2 cells through subcutaneous inoculation in the right flank on day 42 (2 weeks after the last immunization). (**B**) ELISA of the sera against CH401 peptide and native PhMV. (**C**) Flow cytometry analysis of the sera binding to DDHER2 cancer cells, the mean fluorescence intensity (MIF) of the triplicate experiments and standard deviations are shown. Data were analyzed using the FlowJo v10 software. (**D**) MTT assay of complement-dependent cytotoxicity induced by the sera against DDHER2 cells. Cytotoxicity (%) = 100 (experimental OD/control OD) × 100. Mean cytotoxicity of triplicate experiments and standard deviations are shown. Statistical analysis by one-way ANOVA with Tukey’s test: ns = not significant; * *p* < 0.05; ** *p* < 0.01; *** *p* < 0.001.

Following the successful preparation of the PhMV-CH401 and CpG-PhMV-CH401 vaccine formulations, their immune response after subcutaneous prime-boost immunization (3 injections every 2-weeks) was assessed using female BALB/c mice (Figure 2A). Mice were immunized using PhMV-CH401 or CpG-PhMV-CH401 (100 μg per immunization, *n* = 8) vaccine candidates; three control groups (*n* = 6) were immunized with free CH401 peptide or free CpG-ODN 1585 (each used at 2 μg per immunization) or unmodified PhMV. Sera were collected from all mice prior to first immunization (day 0) and one week after the third immunization (day 35), and then pooled to evaluate the antibody responses. Then tumor challenge studies were performed as described below. After systematic analysis comparing the PhMV-CH401 vs. CpG-PhMV-CH401 formulation, we concluded that loading the TLR9 agonist did not confer any enhancement of efficacy. It is possible that the CpGs were loaded onto the surface and not encapsulated, and therefore, may dissociate in vivo, thus not leading to enhanced immune-stimulation. Therefore, in the following, we only discuss the results of the PhMV-CH401 vaccine candidate and present the data for the CpG-PhMV-CH401 in the supporting information (Figures S2–S4).

The sera collected at day 35 were analyzed for CH401 epitope, as well as VLP carrier-specific antibodies, using ELISA (Figure 2B). Only the PhMV-CH401 group yielded anti-CH401-specific IgG responses; this is as expected, because peptides without adjuvants and carriers are generally not immunogenic and clear quickly. Conjugation of CH401 conferred immunogenicity, which can be explained by the VLPs’ transport, cell uptake, and immunostimulatory properties [24,25]. Either vaccine candidate, PhMV-CH401 or CpG-PhMV-CH401, induced significant anti-CH401 IgG responses with an end-point titer of 12,800. Due to the inherent immunogenicity of the VLPs, it is also anticipated that anti-carrier antibodies are produced over the course of treatment [37]. VLP-specific IgG was assessed using ELISA against the PhMV particles and strong IgG titers were observed with an end-point titer >204,800. Nevertheless, the significantly lower absorbance indicates that the anti-VLP antibodies generated have lower affinity relative to the anti-HER2 antibodies.

Next, we evaluated the ability of the mouse sera to bind cancer cells expressing the HER2 receptor; DDHER2 cells and a flow cytometry protocol was used in Figure 2C. While binding of the sera from animals receiving the vaccine candidates (PhMV-CH401 and CpG-PhMV-CH401) produced a positive signal, sera from the control groups receiving unmodified PhMV also tested positive (Figure S2B), indicating non-specific binding—possibly through Fc receptor interactions [38]. Nevertheless, complement-dependent cytotoxicity (CDC) was confirmed by testing sera from the mice immunized using the vaccine candidates (PhMV-CH401 and CpG-PhMV-CH401), but not the control VLPs (Figure 2D and Figure S2C).

**Figure 3:**
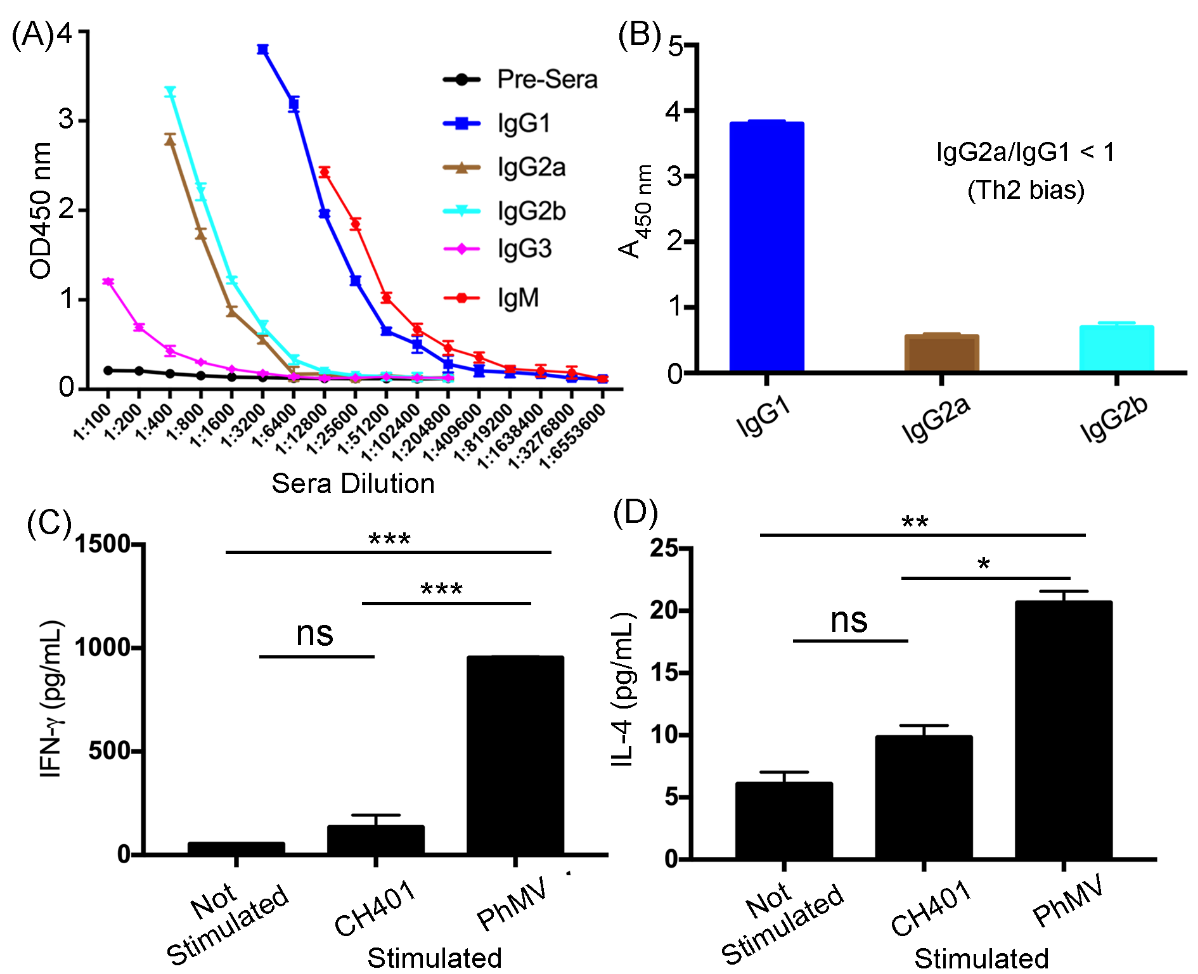
The antibody subtypes and cytokines response from the immunized mice. (**A**) Immunoglobulin subtyping of the sera collected from the PhMV-CH401 group against the CH401 peptide by ELISA. (**B**) IgG1/IgG2a/IgG2b isotypes of the pooled sera at 1:3200 dilution against the CH401 peptide. (**C**) IFN-γ and IL-4 (**D**) secretion from splenocytes isolated from immunized mice, with (stimulated) and without (not stimulated) exposure to CH401 peptide and unmodified PhMV VLPs (20 μg/mL, 18 h). Means of triplicates and standard deviations are shown. Statistical analysis between stimulated and unstimulated cells in each group by two-way ANOVA, with Tukey’s test: ns = not significant; * *p* < 0.05; ** *p* < 0.01; *** *p* < 0.001.

To detail the immunologic response and answer whether the response is Th1 or Th2-biased, IgG isotyping was performed by testing the sera against the CH401 epitope, using ELISA (Figure 3A,B). The free CH401 peptides only induced low IgM responses, confirming that this formulation was only weakly immunogenic (Figure S2A). The PhMV-CH401 stimulated low levels or IgG3, considerable levels of IgG2a and IgG2b, and significantly high levels of the IgM and IgG1 isotypes (Figure 3A). The differences between IgG isotypes suggest distinct immunostimulatory properties of the VLP-based vaccine candidates [25]. The IgG variants bind to Fc receptors and can enhance phagocytosis by macrophages; therefore, each isotype can confer therapeutic efficacy [39,40,41]. Further, IgG1 can fix and mediate antibody-dependent cell cytotoxicity (ADCC) of cancer cells through natural killer cells [39]; IgG2a/2b have the highest binding affinities to FcγRs and are particularly potent mediator of ADCC by myeloid cells, including neutrophils [40,41]. Our data indeed indicate a high level of anti-CH401 IgG1 subtypes with the IgG1/G2a/2b ratio < 1 indicating a Th2 bias (Figure 3B) [42]. This is consistent with other VLPs-based vaccines, for example PVX [25], CCMV and SeMV [27], and influenza VLPs [43] both induce Th1 and Th2 responses, however, with a Th2-dominant responses (in Balb/C mice). Even though the Th1-type response plays an important role in anti-tumor immunity, studies have shown that the dual Th1/Th2 phenotype is desirable in a tumor immunotherapy setting, due to the synergistic roles in combating tumor growth [44]. Antibody subtyping was further corroborated by splenocyte assays and staining for cytokine IFN-γ (Th1) and IL-4 (Th2). Splenoctyes were harvested 7 days post the final immunization, and the cytokines were assayed by culturing splenocytes, with or without the CH401 peptide or unmodified PhMV VLPs. When stimulated with CH401 or PhMV, splenocytes released IFN-γ and IL-4, with PhMV stimulation resulting in stronger responses—and this was consistent with the higher antibody titers produced against the carrier vs. target antigen.

### 3.3. Evaluating the Vaccine Efficacy in a Mouse Tumor Challenge Model

**Figure 4:**
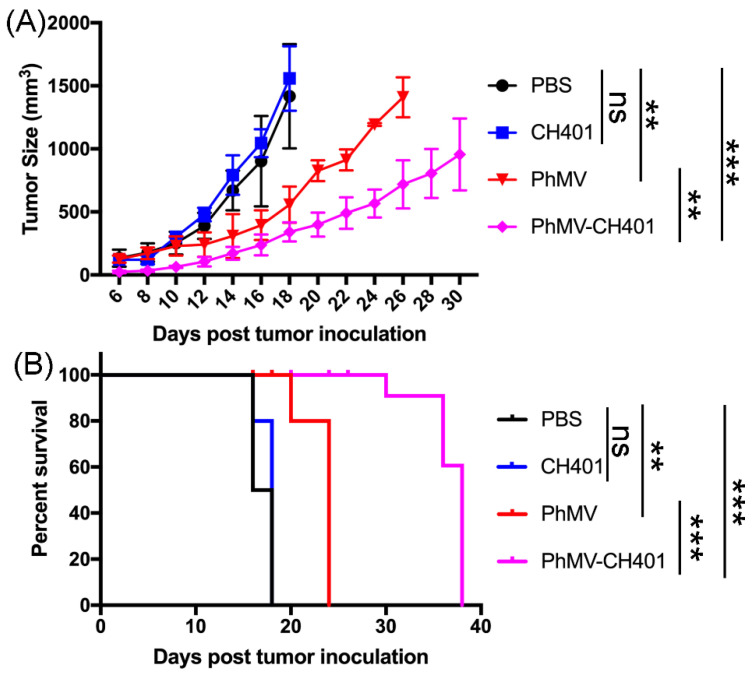
PhMV-based vaccine anti-tumor efficacy in DDHER2 murine model. (**A**) Mean tumor size of mice per treatment group (*n* = 5), statistical analysis was carried out by two-way ANOVA. Mean tumor volumes and standard errors of the mean are shown. (**B**) Statistical analysis of survival curves: log-rank (Mantel–Cox) test. ns = no statistical significance, ** *p* < 0.01; *** *p* < 0.001. (The treatment schedule is shown in Figure 2).

Based on the validated anti-HER2 antibody response from the PhMV-based vaccines, the therapeutic potential was investigated in vivo in a DDHER2 murine model. The mice were subcutaneously inoculated with DDHER2 cells in the right flank (1 × 10^6^ cells/mouse). Tumor volume was monitored daily, once palpable tumors were observed. Tumor progression was very fast in the PBS and free CH401 control groups with tumors reached the endpoint (1000 mm^3^), 14 days post the tumor challenge (Figure 4A). In stark contrast, the group that received the PhMV-CH401 vaccine, exhibited significant delay in tumor growth, resulting in prolonged survival with tumors reaching the endpoint only after 30+ days post the tumor challenge. The efficacy was consistent with antibody titers and CDC (Figure 2 and Figure 3). Interestingly, mice receiving PhMV alone showed modest delay in tumor growth, which may reflect innate immune activation in these mice. In fact, we observed similar effects in our previous study using PhMV as a drug carrier. In these studies, the PhMV group alone also conferred some degree of anti-tumor efficacy [30,31]. We also noted non-specific binding of anti-PhMV sera to DDHER2 cells (Figure 2 and Figure 3), which requires further investigation. Survival data were analyzed using the log-rank (Mantel–Cox) test; all mice in the PhMV and PhMV-CH401 vaccination groups remained alive, when the last mouse in the PBS and CH401 control groups reached their endpoint (Figure 4B). However, the PhMV group only prolonged survival from 17 to 24 days, while immunization with the anti-HER2-specific vaccine candidate PhMV-CH401 led to survival for 38 days post tumor challenge. This result indicates that the secretion of Th1/Th2 cytokines can promote the activity of CD4^+^/CD8^+^ T cells and natural killer cells, thus, helping to prevent tumor growth [45]. Our results, therefore, suggest that PhMV makes a promising platform to develop cancer vaccines with the PhMV-CH401 vaccine candidate inducing effective in vivo protection against the HER2+ cancer.

## 4. Conclusions

HER2+ breast cancers are aggressive tumors and there is a need to develop effective therapeutic and prophylactic vaccine and immunotherapy approaches. Here, we developed two vaccine candidates, PhMV-CH401 and CpG-PhMV-CH401, using the PhMV-based platform nanotechnology. Our data demonstrated potency of the VLP-based HER2 vaccine candidates to induce a strong anti-HER2 immune response, including high anti-CH401 sera, which induced CDC in HER2+ tumor cells. Efficacy of the cancer vaccine candidates was demonstrated using a tumor challenge model using DDHER2 cells and BALB/c mice. The studies demonstrated that vaccination with PhMV-CH401 and CpG-PhMV-CH401 conferred prolonged survival. This study supports the further development of PhMV-based vaccine candidates.

## Data Availability

The data presented in this study are available in this article (and supplementary material).

## Supplementary Materials

The following are available online at https://www.mdpi.com/article/10.3390/cancers13122909/s1. Figure S1: GelRed^TM^-stained agarose gel electrophoresis, Figure S2: Systematic analysis comparing the PhMV-CH401 vs. CpG-PhMV-CH401 formulation through immunological assessment, in a mouse tumor challenge model, Figure S3: Systematic analysis comparing the PhMV-CH401 vs. CpG-PhMV-CH401 formulation through immunoglobulin subtyping analysis, in a mouse tumor challenge model, Figure S4: Systematic analysis comparing the PhMV-CH401 vs. CpG-PhMV-CH401 formulation through vaccine efficacy, in a mouse tumor challenge model.
