# Supplementary material for: Development of a Virus-Like Particle-Based Anti-HER2 Breast Cancer Vaccine
Source: Cancers (Basel). 2021 Jun 10;13(12):2909. doi: 10.3390/cancers13122909 (PMC8230452; doi:10.3390/cancers13122909)
Supplement: Supplementary file 1 [file cancers-13-02909-s001.zip › cancers-1218978-supplementary.pdf]

cancers

MDPI

Supplementary Files

Development of a Virus-Like Particle Based Anti-HER2 Breast Cancer Vaccine

He Hu, Nicole F. Steinmetz

Detailed methods

UV/Visible Spectroscopy

A NanoDrop 2000 spectrophotometer (Thermo Fisher Scientific, Waltham, MA, USA) was used to characterize the UV/vis spectra of native and modified VLPs. Further, protein concentration was measured using the Pierce BCA assay and a Tecan Infinite M200 plate reader.

Native and Denaturing Gel Electrophoresis

Native and modified VLPs (8–10 µg per lane) were analyzed by 1% (w/v) agarose native gel electrophoresis in 0.1 M Tris-maleate running buffer (pH = 6.5). Denatured protein subunits (~10 µg per lane) were analyzed by polyacrylamide gel electrophoresis using 4–12% NuPAGE gels in 1× MOPS buffer (Invitrogen). Samples were denatured by boiling in SDS loading dye for 10 min. Gels were photographed under UV or white light before staining with Coomassie Brilliant Blue, and under white light after staining, using an AlphaImager system (ProteinSimple).

Size Exclusion Chromatography (SEC)

Native and modified VLPs were loaded onto a Superose-6 column on the ÄKTA Explorer system (GE Healthcare). The column was loaded with 100-µL samples (1 mg/mL) at a flow rate of 0.5 mL/min in potassium phosphate buffer pH 7.0.

Transmission Electron Microscopy (TEM)

Native and modified VLPs suspended at 1 mg/mL in 10 µL potassium phosphate buffer pH 7.0 were deposited onto Formvar carbon-coated copper grids (Electron Microscopy Sciences, Hatfield, PA, USA) for 2 min at room temperature. The grids were then washed twice with deionized water for 45 s and stained twice with 2% (w/v) uranyl acetate in deionized water for another 30 s. A Tecnai Spirit G2 transmission electron microscope was used to analyze the samples at 80 kV.

Supporting Data:

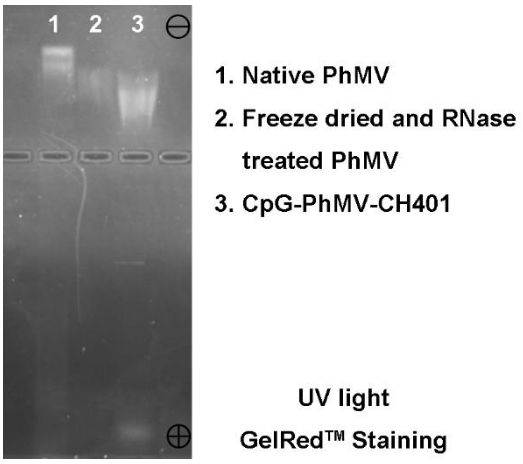

Figure S1. Agarose gel electrophoresis of VLPs to determine whether or not the host RNA of PhMV VLPs was removed. After electrophoretic separation, agarose gels were stained with GelRed™ and then the gels were imaged under UV light; RNA-containing PhMV is detectable (Lane 1), only trace amount of RNA is detectable in freeze dried and RNase A-treated PhMV (Lane 2 suggesting removal of the VLP's nucleic acids); the CpG-ODN 1585 is detected after loading suggesting loading or adsorption into or onto PhMV VLPs.

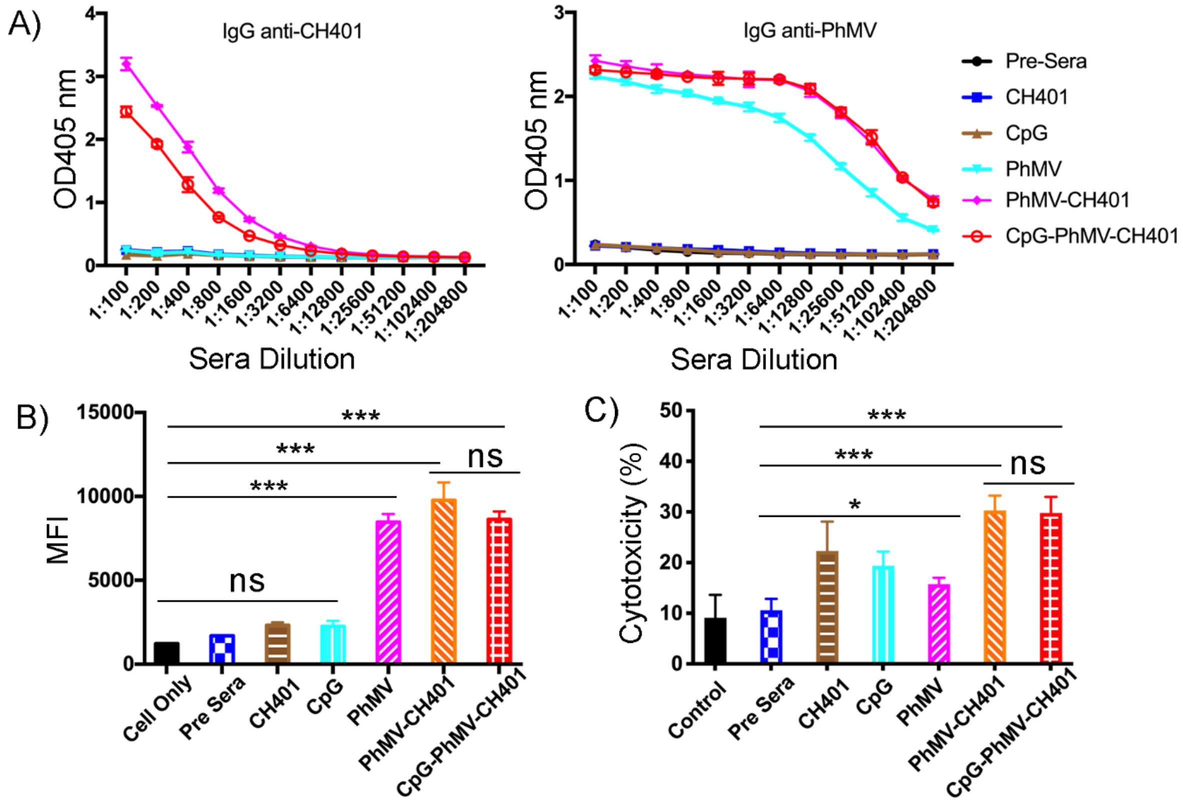

Figure S2. Systematic analysis comparing the PhMV-CH401 vs. CpG-PhMV-CH401 formulation through immunological assessment, in a mouse tumor challenge model. (**A**) Sera were collected before and after immunization of female Balb/c mice and IgG were analyzed by ELISA against CH401 peptide and native PhMV. (**B**) Flow cytometry analysis of the sera binding to DDHER2 cancer cells; the mean fluorescence intensity (MIF) of triplicate experiments and standard deviations are shown. Data were analyzed using FlowJo v10 software. (**C**) MTT assay of DDHER2 to assays for complement dependent cytotoxicity induced by the sera. Cytotoxicity (%) = 100−(experimental OD/control OD) × 100. Mean cytotoxicity of triplicate experiments and standard deviations are shown. Statistical analysis by one-way ANOVA with Tukey's test: *ns* = not significant; * $p < 0.05$; ** $p < 0.01$; *** $p < 0.001$.

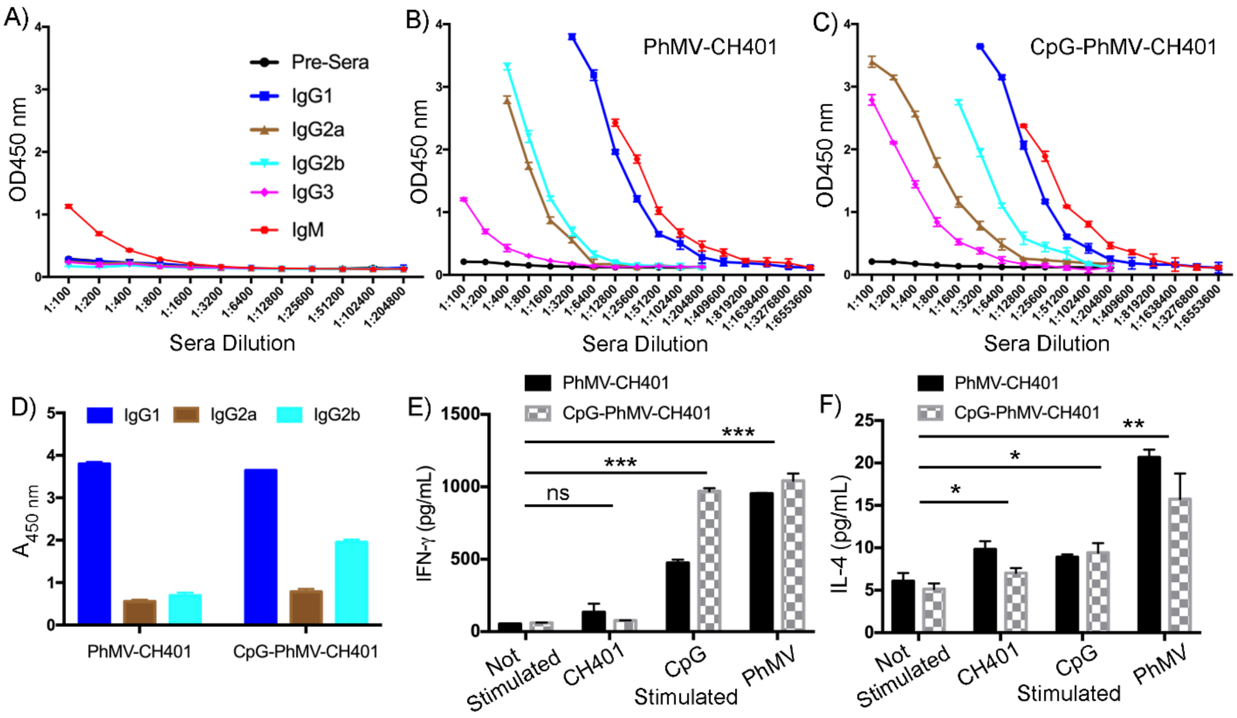

Figure S3. Immunoglobulin subtyping of the sera collected from (**A**) free CH401, (**B**) PhMV-CH401 and (**C**) CpG-PhMV-CH401 group against the CH401 peptide by ELISA. (**D**) The IgG1/IgG2a/IgG2b isotypes of the pooled sera at 1:3200 dilution. (**E**) IFN-γ and IL-4 (**F**) secretion from splenocytes isolated from immunized mice with (stimulated) and without (not stimulated) exposure to CH401 peptide, CpG-ODN 1585 and native PhMV particles (20 µg/mL,18 h) stimulation in vitro, respectively. Means of triplicates and standard deviations are shown. Statistical analysis between stimulated and unstimulated cells in each group by two-way ANOVA with Tukey's test: *ns* = not significant; * $p < 0.05$; ** $p < 0.01$; *** $p < 0.001$.

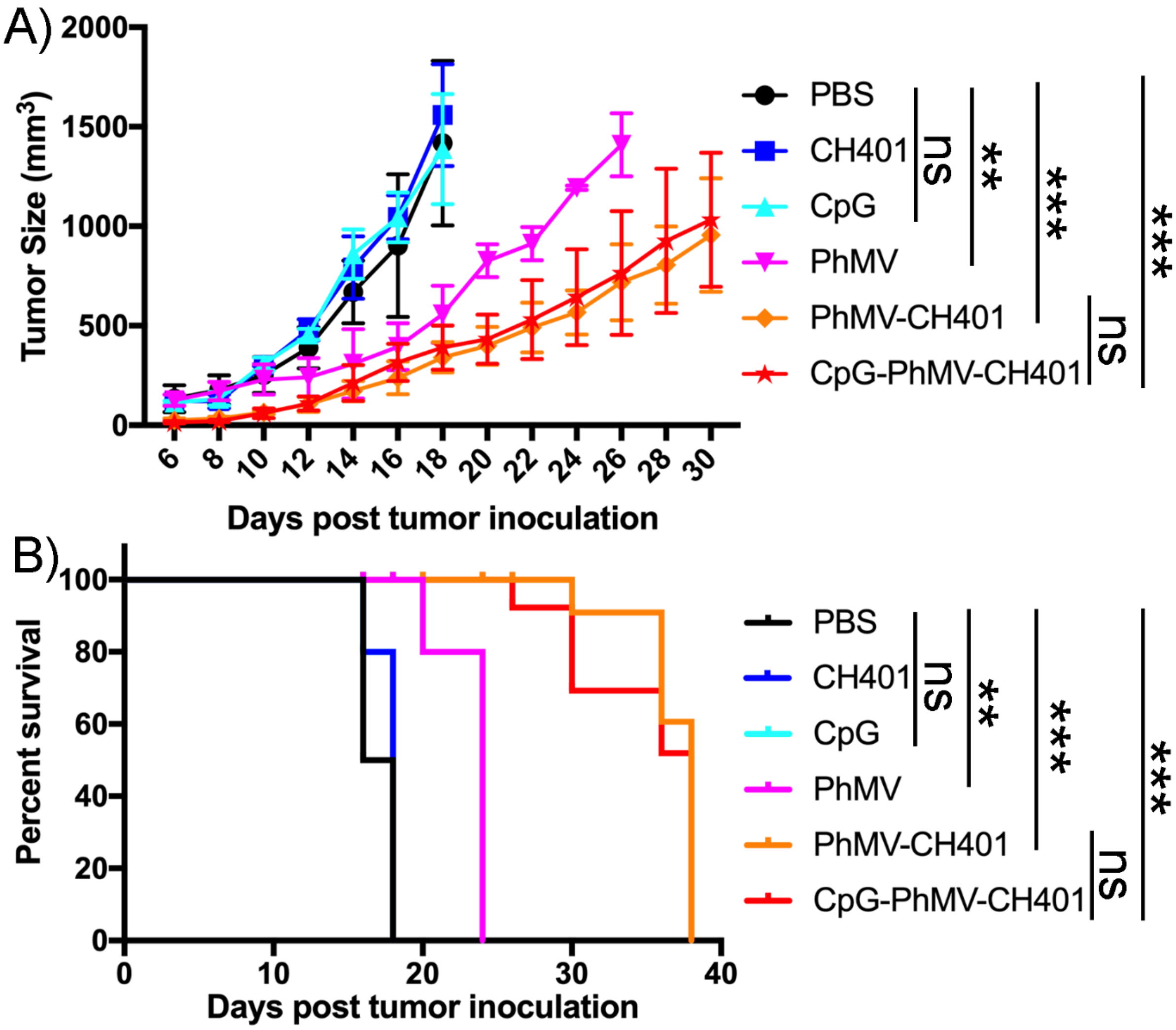

Figure S4. Systematic analysis comparing the PhMV-CH401 vs. CpG-PhMV-CH401 formulation through vaccine efficacy, in a mouse tumor challenge model. (**A**) Mean tumor size of all mice of each group (n = 5), Statistical analysis was carried out by two-way ANOVA. Mean tumor volumes and standard errors of the mean are shown. (**B**) Statistical analysis of survival curves: log-rank (Mantel-Cox) test. *ns* = no statistical significance, ** $p < 0.01$; *** $p < 0.001$.
